# A Rare Case of Retrogastric Abscess Occurring Six Months after N-Butyl-2-Cyanoacrylate Injection into Gastric Varices

**DOI:** 10.1155/2018/7028578

**Published:** 2018-01-08

**Authors:** Ikram Hussain, Andrew Eu Boon Kwek, Veeraraghavan Meyyur Aravamudan, Chern Hao Chong, Tiing Leong Ang

**Affiliations:** ^1^Gastroenterology and Hepatology, Woodlands Integrated Health Campus (WIHC), Singapore; ^2^Gastroenterology and Hepatology, Changi General Hospital, Singapore; ^3^Department of Medicine, Woodlands Integrated Health Campus (WIHC), Singapore

## Abstract

*Background*. Injection with N-butyl-2-cyanoacrylate is a proven and successful therapeutic modality for treatment of patients with bleeding gastric varices. However, a variety of complications have also been associated with its use. Here, we report a rare case of retrogastric abscess which occurred almost six months after this therapy. This abscess was attributed to the hampered microbial clearance caused by the venous obliterations from N-butyl-2-cyanoacrylate. The abscess was successfully treated with 3 months of antibiotics.

## 1. Introduction

Gastrointestinal (GI) variceal bleeding due to portal hypertension is one of the common medical emergencies. Its therapy begins with adequate resuscitation with fluids and splanchnic vasoconstrictors (e.g., somatostatin or terlipressin) and eventually requires an urgent upper GI endoscopy [1]. Upon endoscopy, most patients are found to harbour esophageal varices while in a minority of patients, gastric varices are the primary sites of bleeding. The hemorrhage due to gastric varices is usually more profuse and life-threatening as compared to the bleeding from esophageal varices. Successful endoscopic hemostasis of such bleeding gastric varices can be technically challenging, and a variety of modalities are often tried. In countries where it is approved, N-butyl-2-cyanoacrylate (Histoacryl®; B, Braun, Melsungen, Germany) intravariceal injection therapy is considered the first line therapy to obliterate bleeding gastric varices [1]. This chemical, upon coming in contact with blood or water, quickly polymerises into a solid mass which then clogs the gastric varices. The widespread utilization of such practice is supported by several studies claiming the efficacy of such therapy to be over 80%–90% [2, 3].

Although efficacious in halting gastric variceal hemorrhage, the use of N-butyl-2-cyanoacrylate has been associated with embolic or/and infective complications. The latter complication is thought to result from the break in the mucosa along with introduction of foreign materials [4, 5]. In the majority of such cases, complications related to the infection are self-resolving and without any consequences, though serious infections have infrequently been reported. Here, we report a rare and late-occurring case of retrogastric abscess after injection of N-butyl-2-cyanoacrylate into the bleeding gastric varices.

## 2. Case Report

**Figure 1:**
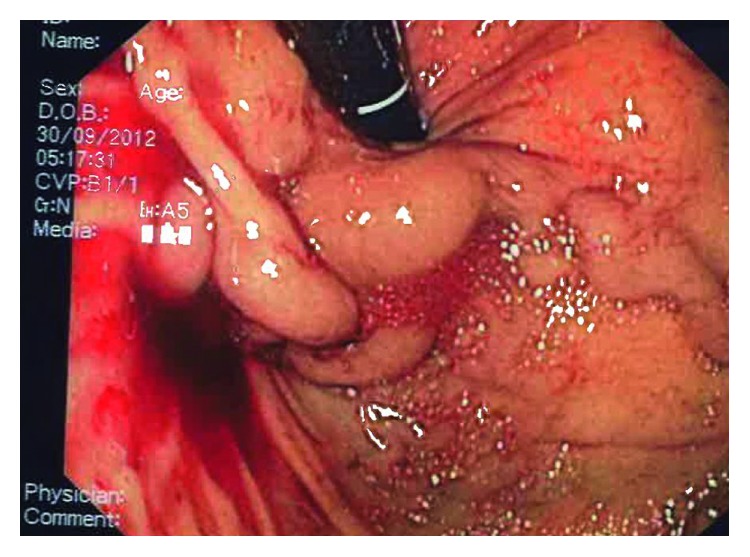
Endoscopic image of bleeding varices in the cardia and fundus.

**Figure 2:**
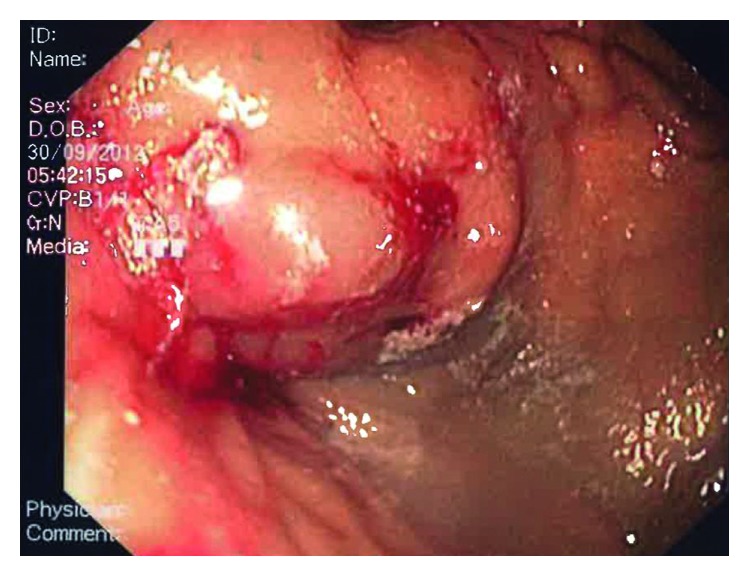
Endoscopic image of hardened gastric varices after intravariceal injection with N-butyl-2-cyanoacrylate.

A 39-year-old Chinese man was hospitalized for right thigh cellulitis. He had a past medical history of poorly controlled type 2 diabetes mellitus. Based on clinical, biochemical, and radiological assessment, he was also concomitantly diagnosed with Child's C liver cirrhosis which was attributed to alcoholic liver disease. The values of initial laboratory tests are represented in Table 1. The ultrasonography of abdomen revealed small amount of ascites and patent portal vein. On day 9 of hospitalization, the patient suffered a massive hematemesis. After adequate resuscitation, an urgent upper GI endoscopy was performed by an experienced procedurist. During endoscopy, gastroesophageal varices (Sarin's GOV-2) with large gastric component (at the cardia and fundus) toward the greater curvature were visualized (Figure 1). A white spot, the “nipple sign,” was visible at one of the gastric varices, suggesting it to be the point of hemorrhage. The gastric varices were injected with the commercially prepared mixture of the N-acetyl-2-cyanoacrylate and Lipiodol (Laboratoire Guerbet, Aulnay-sous-Bois, France) in 0.5 ml : 0.8 ml ratio. The procedurist and nurses adhered to the institutional protocol of intravariceal injection of N-acetyl-2-cyanoacrylate. A total of 5 ml of the mixture was injected in 5 separate aliquots at 5 different locations with complete obliteration (Figure 2). Additionally, three variceal ligation bands were applied to the varices in the lower esophagus. Although prophylactic antibiotic therapy (intravenous ceftriaxone) was administered, patient developed fever five days after the variceal treatment. This fever was attributed to a new urinary tract infection (UTI) with growth of pan-sensitive *Escherichia coli* in urine. A computed tomography of the abdomen was performed which again showed small amount of ascites, but no localized intra-abdominal collection was observed. Additionally, radiological appearances of the pancreas were unremarkable. The patient was discharged well after treatment of the UTI. Nonselective beta-blocker therapy (tablet propranolol) was initiated before discharge. The patient was advised to complete the courses of antibiotics (namely, five more days of cloxacillin for cellulitis and ten days of ciprofloxacin for UTI).

**Figure 3:**
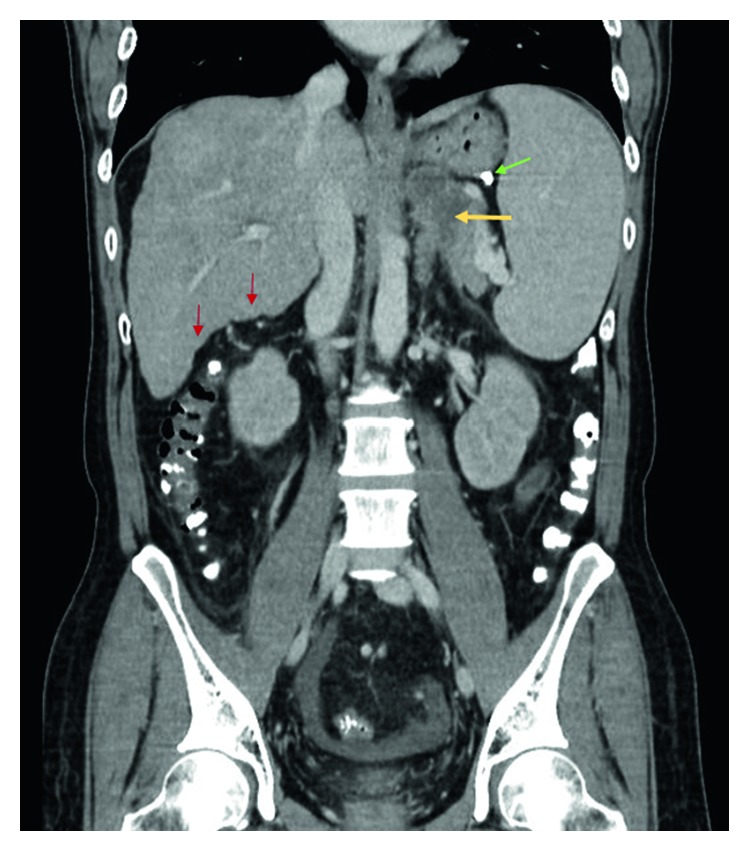
Computed tomography view of the abdomen (in coronal view). The liver contour is nodular (red arrows) confirming the presence of cirrhosis. There is a hypodense collection (yellow arrow) near the splenic hilum and below the stomach, corresponding to the retrogastric abscess. The small amount of residual N-butyl-2-cyanoacrylate is brightly visible in the perigastric region (green arrow).

**Figure 4:**
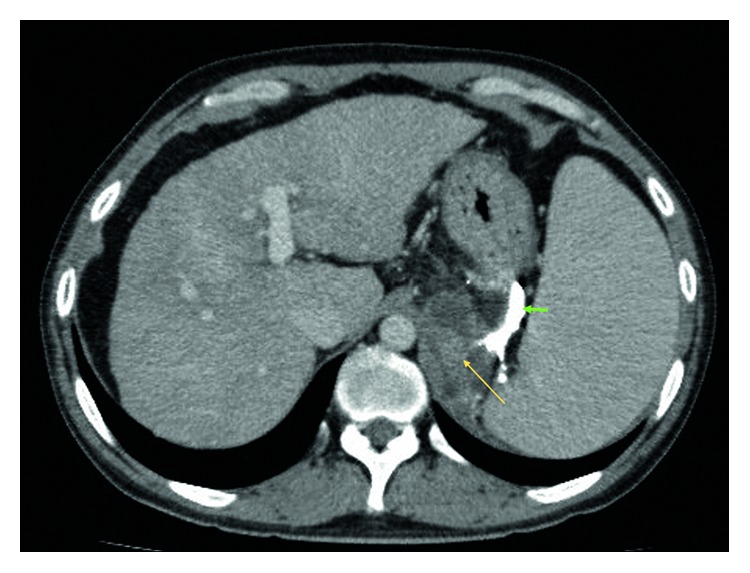
Computed tomography view of the abdomen (in axial view). The hypodense collection (yellow arrow) behind the stomach corresponds to the retrogastric abscess. The residual N-butyl-2-cyanoacrylate in the perigastric region is more explicit in this view (green arrow).

**Figure 5:**
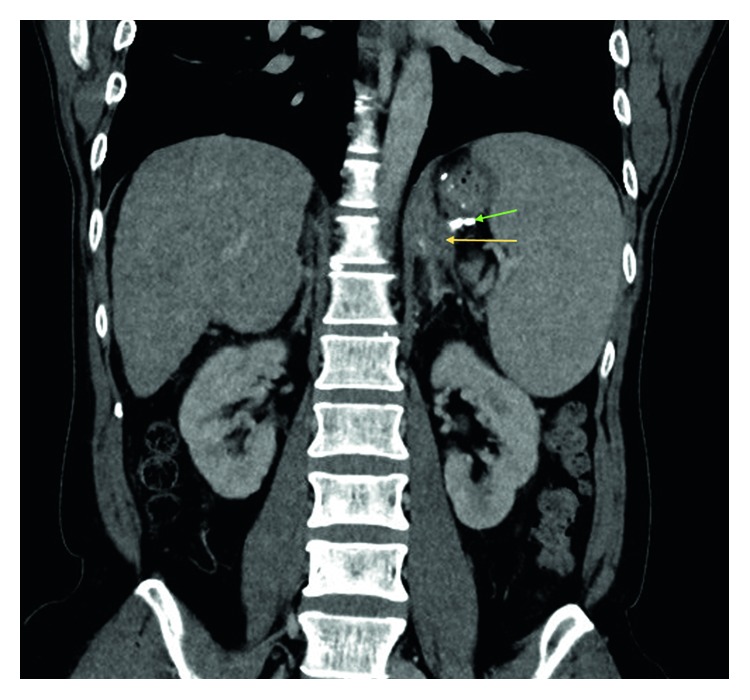
Computed tomography view of the abdomen (in coronal view) after completion of antibiotics. The previously seen retrogastric abscess has completely resolved leaving behind a small area of scarred tissue (yellow arrow). The residual N-butyl-2-cyanoacrylate in the perigastric region is still visible (green arrow).

**Figure 6:**
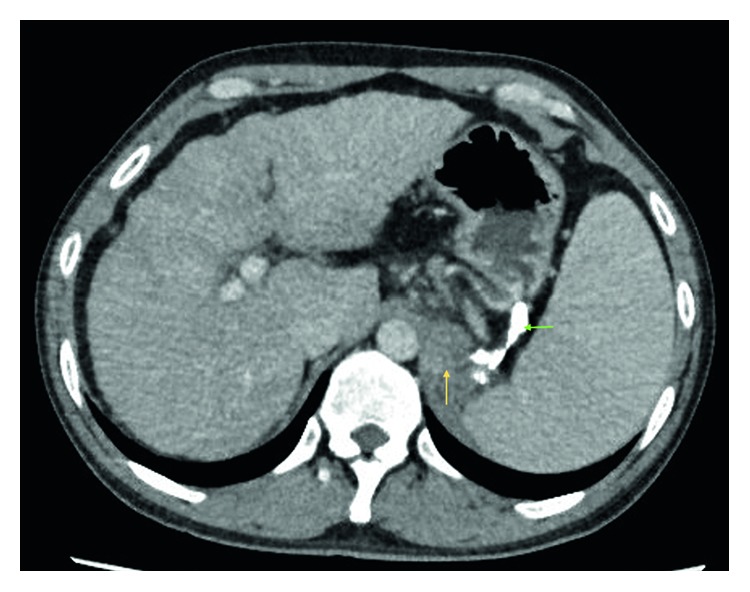
Computed tomography view of the abdomen (in axial view) after completion of antibiotics. The previously seen retrogastric abscess has completely resolved leaving behind a small area of scarred tissue (yellow arrow). The residual N-butyl-2-cyanoacrylate in the perigastric region is still visible (green arrow).

A follow-up endoscopy was performed after 10 days of the index endoscopy where complete obliteration of the gastroesophageal varices was ascertained. The patient remained well for next 6 months until he was rehospitalized for high spiking fever. He did not report any epigastric pain. The abdomen was soft and not tender. The patient had been abstinent from alcohol for last 6 months. The values of initial laboratory tests are shown in Table 1. Parenteral antibiotic therapy (ceftriaxone) was administered. Growth of pan-sensitive *Escherichia coli* was noted in the aerobic blood culture. A computed tomography (CT) of the abdomen and pelvis was performed in view of nonresolving fever despite clearance of bacteremia in subsequent blood cultures. The CT scan (Figures 3 and 4) showed a retrogastric abscess (5.1 cm × 3.4 cm × 4.3 cm in size) adjacent to the left diaphragmatic crus and pancreatic tail. The previously injected N-butyl-2-cyanoacrylate was still visible around the gastric cardia, in the perigastric varices, and in the left adrenal vein. Also, there was a new partial thrombosis of the left renal vein. As compared to the abdominal imaging six months ago, there was no ascites. Additionally, there were no radiological features of acute or chronic pancreatitis. An infectious disease specialist was consulted who suspected inadequate penetration of the abscess with cephalosporin. The antibiotic therapy was switched to carbapenem, and a conservative nondrainage approach was undertaken for two reasons: the bacteremia had already cleared, and the size of the abscess was relatively small for drainage. Another CT scan was performed after one week which confirmed interval reduction in the size of retrogastric abscess (now 4.8 cm × 2.4 cm × 4.5 cm). After clinical improvement, the patient was subsequently discharged thirteen days after hospitalization. Further antimicrobial therapy was administered at the outpatient parenteral therapy centre. Two CT scans (Figures 5 and 6) were repeated at one month intervals, which showed gradual resolution of the retrogastric abscess to a small nonliquid lesion. Overall, the patient received three months of antibiotics. Over next one year of follow-up, there was no clinical or radiological recurrence of the retrogastric abscess.

## 3. Discussion

Gastrointestinal variceal bleeding is a frequently encountered medical emergency in acute care hospitals, and endoscopic hemostatic therapy is emergently employed in such scenario. Bleeding gastric varices are found in a minority of such patients. As compared with the esophageal varices, the incidence of hemorrhage from gastric varices is lower, but the bleeding from the latter tends to be more severe, more difficult to control, and more life-endangering. Intravariceal injection of gastric varices with tissue adhesives (or glues), for example, N-butyl-2-cyanoacrylate, is considered more effective than sclerotherapy or variceal ligation [2, 3]. Although the injection of N-butyl-2-cyanoacrylate is considered effective, many complications have been reported with its use, namely, thromboembolism and septic complications [6, 7]. For instance, Ausloos et al. recently enumerated 9 cases of N-butyl-2-cyanoacrylate glue-related infection in the literature [8]. To our knowledge, there has been only one case reported before our case of a retrogastric abscess after cyanoacrylate variceal injection [9].

The septic complications associated with the injection of N-butyl-2-cyanoacrylate are attributed to a variety of factors: break in the mucosal barrier with the injection or with the posttherapeutic mucosal ulceration; deposition of foreign material; and reduced antimicrobial clearance in clogged blood vessels. Although prophylactic antibiotics are administered while treating bleeding varices, delayed serious septic complications may still occur due to the aforementioned reasons. Lee et al. reported two cases of delayed adrenal abscess which happened 4–6 months after the variceal injections [10]. Similarly, in our case, the retrogastric abscess happened 6 months after variceal injection with N-butyl-2-cyanoacrylate. We believe that the abscess had occurred due to persistent deposition of N-butyl-2-cyanoacrylate which acted as a nidus for microbes and also hindered clearance of microbes due to adjacent venous obliterations. As we did not aspirate the collection, confirmation of *Escherichia coli* in its content could not be performed. Although this is a limitation in the present case, the retrogastric collection behaved like an abscess in the clinical context and thus responded to antimicrobial therapy.

This case highlights a rare complication of retrogastric abscess which occurred six months after gastric variceal injection with N-butyl-2-cyanoacrylate. As the size of abscess was small in our patient, nondrainage antimicrobial management was successful. Larger abscesses or poor responders should be considered for drainage as the penetration of antimicrobials may be inadequate. Although abscess formation is a rare occurrence after variceal injection with N-butyl-2-cyanoacrylate, our report emphasizes to consider the differential diagnosis of a perigastric abscess in a septic patient, even if the use N-butyl-2-cyanoacrylate was in the past.

## Figures and Tables

**Table 1:** Values of initial laboratory tests during the two respective hospitalizations.

Test	Time A	Time B	Unit	Reference interval
White blood cells	12.32	7.42	×10^9^/L	3.40–9.60
Red blood cells	3.1	2.90	×10^12^/L	3.70–9.60
Hemoglobin	8.7	8.1	g/dL	10.9–15.1
Mean cell volume	91.5	89.3	fL	80.0–95.0
Mean corpuscular hemoglobin	30.6	27.9	pg	27.0–33.0
Mean corpuscular hemoglobin concentration	34.1	33.8	g/dL	32.0–36.0
Hematocrit	33.5	31.2	%	32.7–44.4
Platelets	112	126	—	132–372
Mean platelet volume	10.1	9.7	—	8.7–12.2
Red cell distribution width	14.1	13.7	%	11.4–14.8
Sodium	133	134	mEq/L	135–145
Potassium	3.6	4.4	mEq/L	3.5–5.0
Chloride	95	97	mEq/L	95–110
Carbon dioxide	26	22	mEq/L	22–31
Creatinine	1.19	1.39	mg/dL	0.57–1.02
Urea	25.49	21.84	mg/dL	5.60–18.21
Glucose	176.6	142.3	mg/dL	72.0–140.5
Albumin	2.3	3.0	g/dL	3.8–4.8
Bilirubin, total	3.16	1.57	mg/dL	0.29–1.75
Bilirubin, conjugated	2.57	1.29	umol/L	0–0.29
Aspartate aminotransferase	115	98	U/L	10–50
Alanine aminotransferase	69	43	U/L	10–70
Alkaline phosphatase	141	126	U/L	40–130
Prothrombin time	14.4	14.3	Seconds	9-2-11

Time A denotes the period when variceal bleeding occurred, and Time B represents the hospitalization for retrogastric abscess.
